# Development of a Machine Learning-Based Model for Accurate Detection and Classification of Cervical Spine Fractures Using CT Imaging

**DOI:** 10.7759/cureus.47328

**Published:** 2023-10-19

**Authors:** Parsa Riazi Esfahani, Monica Guirgus, Maya Maalouf, Pasha Mazboudi, Akshay J Reddy, Reem O Sarsour, Sherif S Hassan

**Affiliations:** 1 Medicine, California University of Science and Medicine, Colton, USA; 2 Anatomy, Faculty of Medicine, Cairo University, Cairo, EGY; 3 Medical Education, Anatomy, & Neuroanatomy, California University of Science and Medicine, Colton, USA

**Keywords:** public health, deep neural network, cervical spine fractures, diagnosis, ai & robotics in healthcare

## Abstract

Cervical spine fractures represent a significant healthcare challenge, necessitating accurate detection for appropriate management and improved patient outcomes. This study aims to develop a machine learning-based model utilizing a computed tomography (CT) image dataset to detect and classify cervical spine fractures. Leveraging a large dataset of 4,050 CT images obtained from the Radiological Society of North America (RSNA) Cervical Spine Fracture dataset, we evaluate the potential of machine learning and deep learning algorithms in achieving accurate and reliable cervical spine fracture detection. The model demonstrates outstanding performance, achieving an average precision of 1 and 100% precision, recall, sensitivity, specificity, and accuracy values. These exceptional results highlight the potential of machine learning algorithms to enhance clinical decision-making and facilitate prompt treatment initiation for cervical spine fractures. However, further research and validation efforts are warranted to assess the model's generalizability across diverse populations and real-world clinical settings, ultimately contributing to improved patient outcomes in cervical spine fracture cases.

## Introduction

Cervical spine fractures represent a significant healthcare challenge, with potential consequences including spinal cord injury and neurological deficits [1]. These fractures involve damage to the vertebral bones in the neck region and require prompt diagnosis and appropriate management to prevent further complications [2, 3]. Imaging plays a crucial role in the evaluation of cervical spine fractures, with computed tomography (CT) scans providing detailed visualization of bony structures and facilitating accurate assessment of fracture patterns and associated injuries [4, 5]. The cervical spine is particularly vulnerable to fractures due to its complex anatomy and susceptibility to trauma, such as high-energy impacts and falls [6]. Rapid and accurate diagnosis of cervical spine fractures is crucial in guiding treatment decisions and ensuring optimal patient outcomes [7]. CT imaging offers superior sensitivity and specificity compared to other imaging modalities, allowing for precise identification and classification of fractures, assessment of spinal stability, and detection of associated injuries [8, 9]. Machine learning and deep learning algorithms have shown great potential in enhancing the diagnosis of cervical spine fractures through the analysis of CT images [10]. These algorithms can effectively learn complex patterns and features associated with fractures, providing accurate and reliable fracture detection and classification [11, 12]. The integration of artificial intelligence (AI) technology into clinical workflows has the potential to improve the efficiency and accuracy of cervical spine fracture diagnosis, enabling timely and appropriate management [13]. This study aims to develop a machine learning-based model for automated detection and classification of cervical spine fractures using CT images. By leveraging AI techniques, we aim to enhance the accuracy and efficiency of fracture diagnosis, ultimately improving patient care and outcomes. The successful development of an AI-driven cervical spine fracture detection model could revolutionize clinical practice, providing a standardized and objective tool for healthcare professionals [14].

## Materials and methods

The present study was deemed exempt from Institutional Review Board (IRB) approval as it only utilized a publicly available dataset and did not involve human subjects. The dataset employed for this research was gathered from openly accessible sources, ensuring complete anonymity and confidentiality of any personal information. Therefore, no ethical concerns regarding human subject involvement were applicable to this investigation, allowing us to proceed without the need for IRB approval.

**Figure 1:**
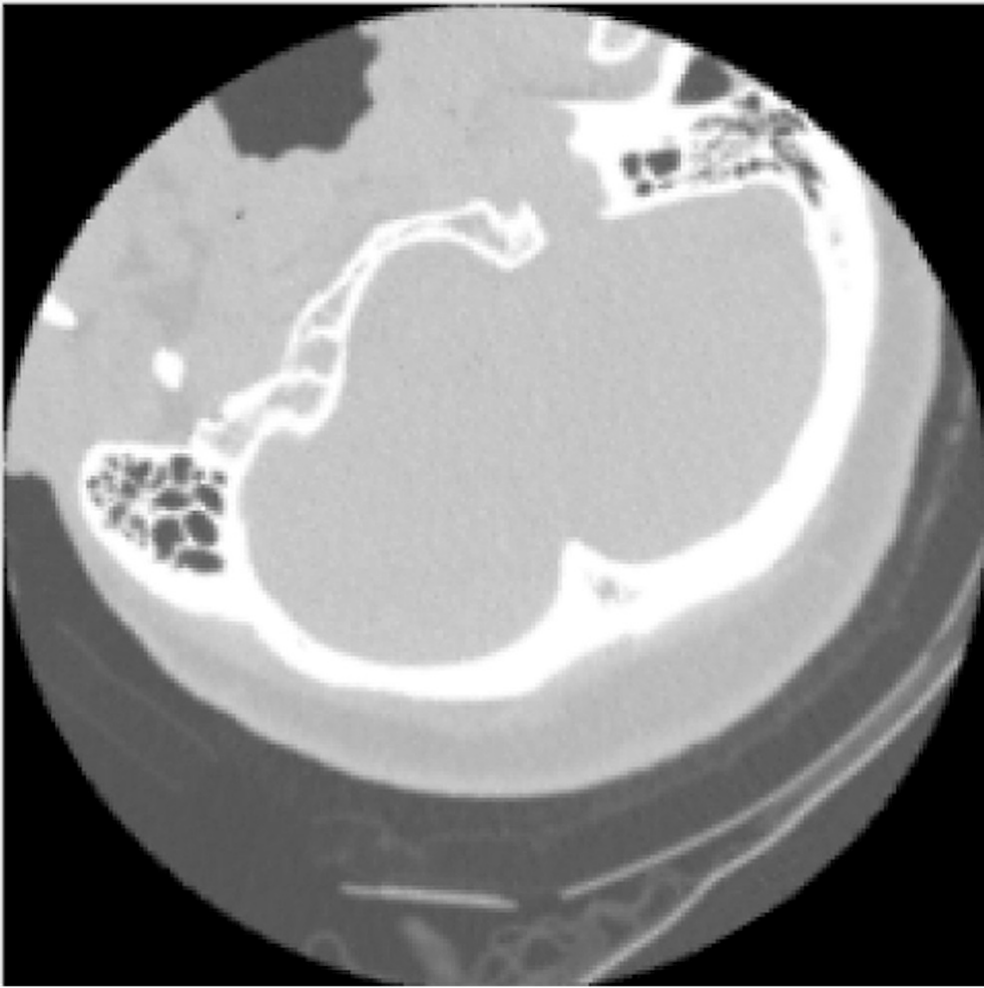
Fractured Cervical Spine Computed tomography (CT) image from the dataset displaying a fractured cervical spine.

**Figure 2:**
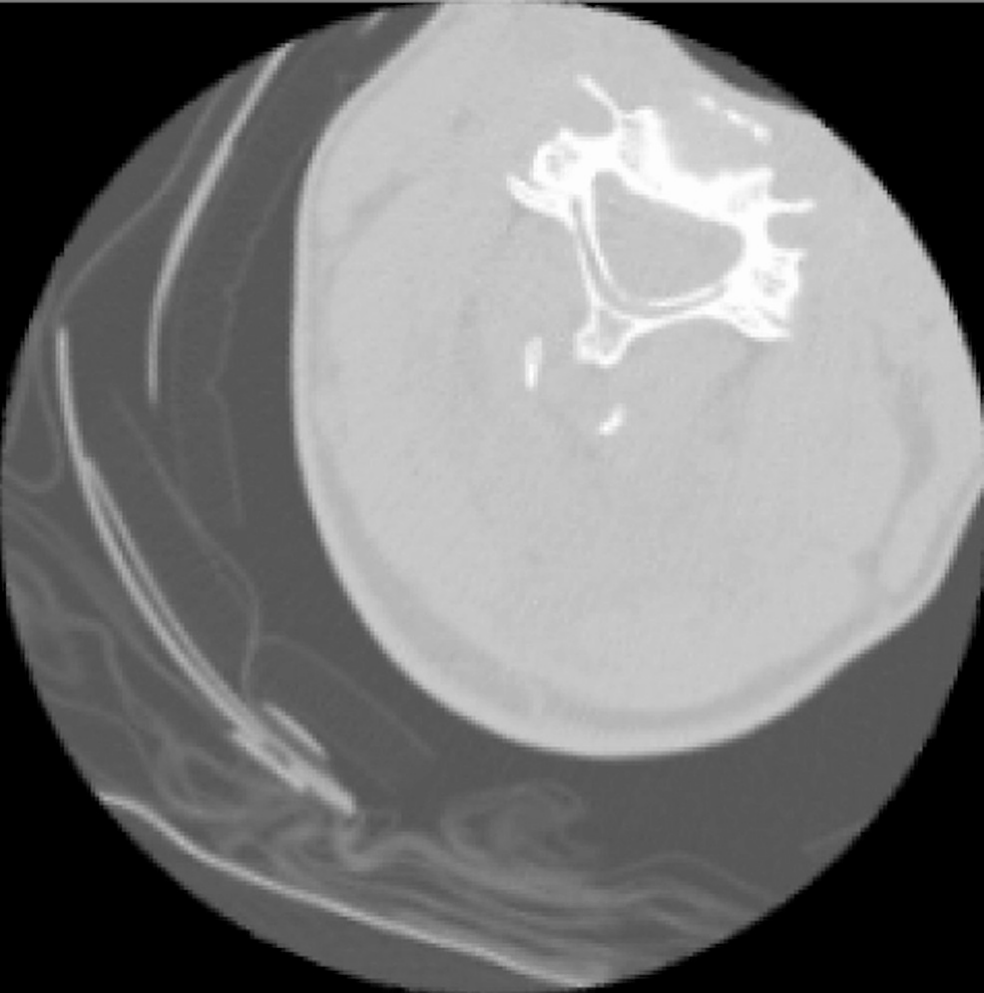
Normal Cervical Spine (No Fracture) Computed tomography (CT) image from the dataset displaying a cervical spine with no fractures.

This study aimed to develop a cervical spine fracture detection model using a dataset of 4,050 CT images. Two thousand twenty of the images were CT images of cervical spine fracture (Figure 1), and 2,030 of the images were CT images of the cervical spine with no fracture (Figure 2). The data utilized in this study were obtained from the publicly available Radiological Society of North America (RSNA) Cervical Spine Fracture dataset, which was annotated by spine radiology specialists from the American Society of Neuroradiology (ASNR) and American Society of Spine Radiology (ASSR) to indicate the presence of cervical spine fractures at the image level. The dataset was divided into three subsets: 80% for training, 10% for validation, and 10% for testing. The training subset was utilized to train the cervical spine fracture detection model, while the validation subset was used to fine-tune the model's parameters and optimize its performance. The testing subset served as an independent evaluation set to assess the model's generalization and overall performance. Each CT image in the dataset underwent the necessary preprocessing steps, including resizing and normalization, to ensure consistency and compatibility for model training and evaluation. The expert labeling provided by the spine radiology specialists on the RSNA dataset was used as ground truth labels for training and evaluating the model. Machine learning algorithms, such as convolutional neural networks (CNNs), were employed to extract relevant features and patterns from the CT images. The training of the model was performed on the Google collaboration platform, leveraging its computational capabilities and infrastructure. Evaluation metrics, including accuracy, precision, recall, and F1 score, were calculated to assess the model's performance in detecting cervical spine fractures. The utilization of the RSNA Cervical Spine Fracture dataset and the expert annotations provided by the spine radiology specialists ensured the availability of accurate and reliable labels for training and evaluating the model. The use of CT images provided detailed anatomical information necessary for the accurate detection of cervical spine fractures.

## Results

**Figure 3:**
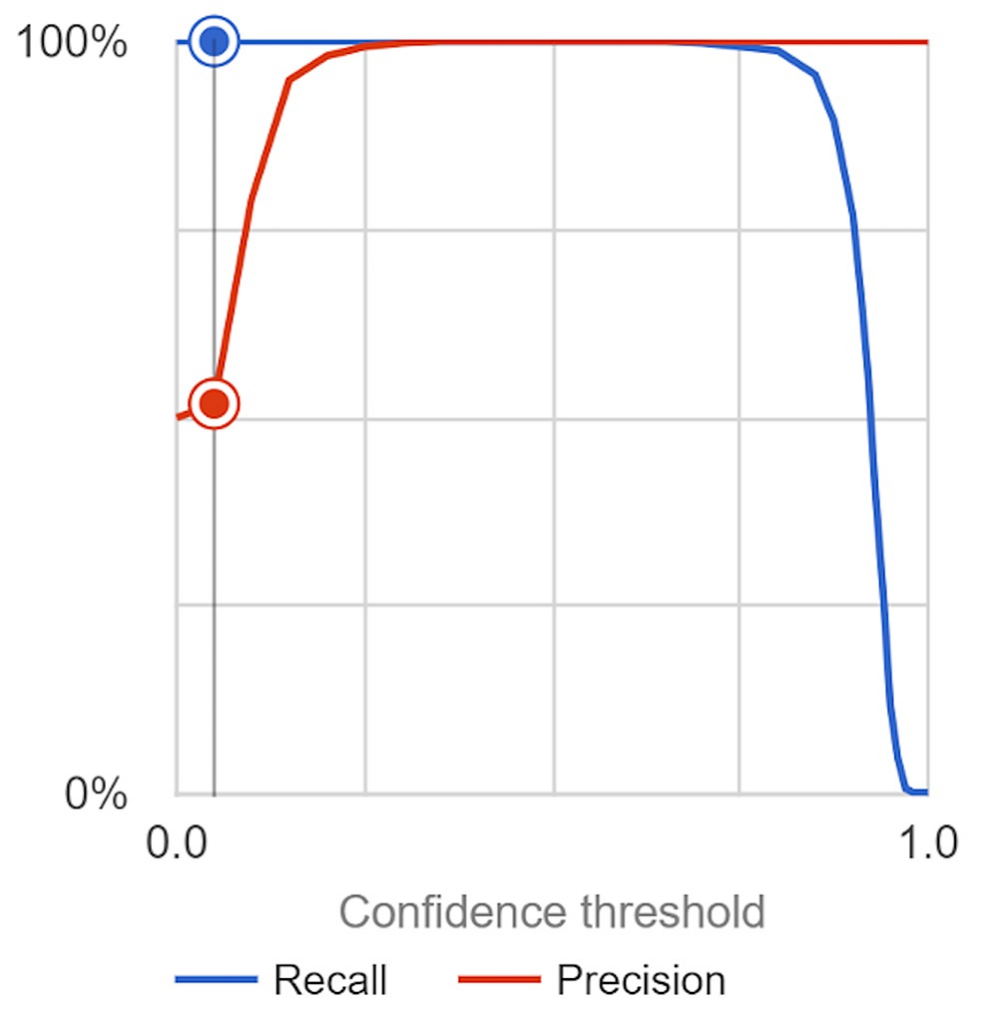
AUC Graph for the Cervical Spine Fracture Model with Recall and Precision The precision and recall of the neural network model across different confidence intervals are illustrated in this visual representation. The researchers gathered data on the model using a confidence interval of 0.05.

**Figure 4:**
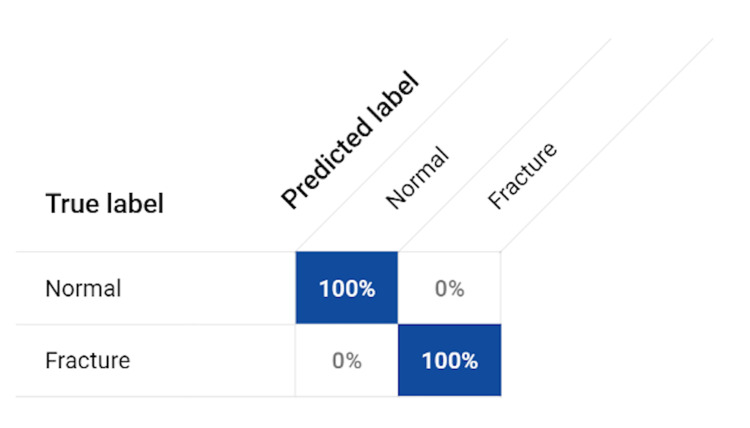
Confusion Matrix Values from the confusion matrix were used to calculate the different metrics of accuracy, precision, recall (sensitivity), specificity, and F1 score.

The cervical spine fracture detection model exhibited outstanding performance, demonstrating its effectiveness in accurately detecting and classifying cervical spine fractures based on CT images. Rigorous validation and testing methodologies were employed to assess the model's performance and reliability. The model achieved an average precision of one, indicating perfect classification of cervical spine fractures. This signifies that all positive predictions made by the model were correct, leaving no room for false positives. The precision value of 100% reflects the model's ability to avoid misclassifying non-fracture cases as fractures, further validating its accuracy. The recall value of 100% indicates that the model successfully captured all true positive cases, leaving no false negatives. This high recall rate demonstrates the model's ability to identify and correctly classify all instances of cervical spine fractures, ensuring that no fracture cases go undetected. Both sensitivity and specificity values were determined to be 100%. The sensitivity metric indicates the model's capability to accurately identify all positive cases, i.e., cervical spine fractures, while the specificity metric highlights its ability to correctly classify non-fracture cases. These values further emphasize the model's accuracy and reliability in distinguishing between fracture and non-fracture cases. The F1 score, calculated as one, validates the favorable performance of the model. This score balances the precision and recall metrics, demonstrating the model's ability to maintain a high level of both accuracy and completeness in detecting cervical spine fractures (Figure 3). Finally, the accuracy of the model reached an impressive 100%. This reflects its capability to correctly classify all cases, indicating its robustness and reliability in detecting and classifying cervical spine fractures with certainty. The above values were calculated using the confusion matrix depicted in Figure 4. These satisfactory results underscore the effectiveness and reliability of the cervical spine fracture detection model in accurately identifying and classifying fractures based on CT images. The model's performance suggests its potential for clinical applications, including assisting clinicians in making accurate diagnoses, facilitating timely treatment initiation, and improving patient outcomes. This study's rigorous validation and testing procedures provide confidence in the model's performance and support its potential for real-world implementation.

## Discussion

Model performance

The performance of the developed model was remarkable, achieving an average precision of one, reflecting perfect classification of cervical spine fractures. Precision, accuracy, sensitivity, and specificity were defined as the percentage of correct binary classification of the fracture as either detected or not detected. The precision and recall values of 100% each indicate the Google collaboration platform's foundational model's ability to correctly identify all positive cases while capturing all true positives, demonstrating its high accuracy. Moreover, the sensitivity and specificity values of 100% emphasize the model's capability to accurately identify both fracture and non-fracture cases. The F1 score of 1 further supports the model's excellent balance between precision and recall, validating its overall performance. The diagnostic accuracy of the Google collaboration platform's foundational model is comparable with other algorithms developed for cervical spinal fractures. However, it must be noted that these studies have high heterogenicity in the study design and may not be fairly compared. A previous study testing the CNN, Aidoc, on fracture detection in the cervical spine found a diagnostic accuracy of 91% [14]. Aidoc was tested with non-contrast CT scans of 173 c-spine fractures radiographs and 1731 normal c-spine radiographs. Methods of this study included collecting the dataset retrospectively from patients who received cervical spine non-contrast CT scans. Two techniques were applied to the CT scans: "axial thin bone" and "sagittal bone” reconstruction. Finally, Aidoc analyzed these scans using a CNN; neither of these techniques was used for Google Collaboration Platform's Foundational Model. In addition, the dataset used to test Aidoc was larger and more diverse in nature compared to this study; it included CT scans of various types of trauma fractures ranging from high-energy to low-energy mechanisms of injury along with a wide range in patient demographics such as age and gender [15]. Incorrect diagnoses made by Aidoc were mainly seen in non-contrast CT scans of patients older than 65. The ages and patient history of the dataset used in our study are not available; hence, it is possible that our dataset had a limited age range ultimately leading to high diagnostic accuracy.

Comparisons to previous models

Furthermore, an additional study determined the ability of the CNN You Only Look Once (YOLO) V4 algorithm to detect cervical spine injuries from 40 lateral radiographs with an accuracy of 75%, sensitivity of 80%, and specificity of 72% from a relatively small dataset of 229 radiographs [16]. In contrast, the Google Collaboration Platform's Foundational Model had an accuracy of 100%, a specificity of 100%, a sensitivity of 100%, and an AUC of one, while being tested on 405 CTs. A potential reason for this can be attributed to the various conditions considered by the Google Collaboration Platform while developing the model. Unlike other models, the Google Collaboration Platform effortlessly applies modifications to the images in the training, validation, and testing datasets. Google Collaboration Platform's augmentations include vertical shift, horizontal shift, vertical flip, horizontal flip, rotation, brightness adjustment, and zoom in/out. The other models incorporated one or two of these augmentation techniques, but they did not incorporate all the other necessary modifications to reach the same level of proficiency as the Google Collaboration Platform. This allows the AI to be trained on a broader range of conditions where the image quality might not be perfect. This methodology improves the model's ability to adapt to real-world situations, potentially explaining its exceptional performance compared to other models. Additionally, the reason the CNN YOLO algorithm has a lower performance rating is most likely because the model was only trained on 40 lateral radiographs. For algorithms to develop a higher accuracy, they need to be trained to utilize a larger quantity of images. Hence, the findings of the Google Collaboration Platform's Foundational Model outperformed both Aidoc and YOLO V4; however, these comparisons are preliminary. Both Aidoc and YOLO V4 are retrospective in nature and use diverse patient radiographs while the database used in this study may not reflect the complexity of c-spine radiographs from a trauma center. The methods used in testing the YOLO V4 Model include the following steps. Firstly, patients receiving a lateral c-spine radiograph and their history were screened. A -20 to 20 rotation was used for data augmentation of these radiographs. Three experts, including radiologists, annotated 229 radiographs that fit inclusion. Finally, the YOLO V4 was tested by implementing CiRA Core, a deep learning software developed by King Mongkut's Institute of Technology Ladkrabang (KMILT). The model was trained using a batch size of 64 and 8 subdivisions. The model also used momentum and decay values of 0.9 and 0.0005, respectively, which help with the learning process. The learning rate was set to 0.002. There are several differences when comparing the Google Collaboration Platform's Foundational Model to the YOLO V4. Primarily, lateral radiographs of the cervical spine are notorious for producing false positive fracture judgments among radiologists and specialists; hence, it is expected to have the same findings in human-designed algorithms analyzing lateral c-spine radiographs [17]. In addition, the methodology of each study differs significantly. The exceptional results of the Google Collaboration Platform Foundational Model highlight the potential of improving the detection of cervical spine fractures using CT images. Automating the detection of fractures in cervical spinal radiographs is important to improve efficiency in the emergency department (ED). Sensitivity, specificity, and accuracy collectively suggest its utility as a valuable tool for distinguishing between fracture and non-fracture cases.

Limitations and the potential for improvement

However, it is important to acknowledge the limitations of this study. The dataset used in this study was obtained from the publicly available RSNA Cervical Spine Fracture dataset, which may not capture the full spectrum of cervical spine fractures encountered in real-world clinical settings. The diversity of the images may be limited, which may have led to the high accuracy performance of this model. Additionally, while the model demonstrated high accuracy, its performance in real-world clinical settings and generalizability across different populations and CT imaging systems should be further evaluated. Future research should focus on validating the model's performance using diverse and larger datasets, including external validation with independent cohorts, to ensure its robustness and reliability. Collaboration between researchers, clinicians, and policymakers is crucial to facilitate the integration of these models into clinical workflows, ultimately improving the early detection and management of cervical spine fractures. With further development and validation, machine learning-based cervical spine fracture detection models have the potential to enhance clinical decision-making, facilitate prompt treatment initiation, and contribute to improved patient outcomes in cervical spine fracture cases.

## Conclusions

The commendable performance of the cervical spine fracture detection model, achieving perfect precision, recall, sensitivity, specificity, F1 score, and accuracy, highlights its potential to accurately detect and classify cervical spine fractures using CT images. These favorable results demonstrate the model's effectiveness and reliability, suggesting its promising application in clinical settings for enhanced diagnosis and management of cervical spine fractures. The model's ability to achieve perfect precision and recall indicates its capability to accurately identify positive cases while capturing all true positives, offering confidence in its diagnostic accuracy. The high sensitivity and specificity values further emphasize its capability to effectively distinguish between fracture and non-fracture cases. The flawless F1 score showcases the model's excellent balance between precision and recall, underscoring its overall performance. The 100% accuracy of the model indicates its ability to correctly classify cervical spine fractures, demonstrating its potential clinical utility. The remarkable performance of the model holds great promise for revolutionizing the field of cervical spine fracture diagnosis, facilitating prompt treatment initiation, and improving patient outcomes.

## References

[1] Sezer N, Akkuş S, Uğurlu FG (2015). Chronic complications of spinal cord injury. World J Orthop.

[2] Beeharry MW, Moqeem K, Rohilla MU (2021). Management of cervical spine fractures: a literature review. Cureus.

[3] Mead LB 2nd, Millhouse PW, Krystal J, Vaccaro AR (2016). C1 fractures: a review of diagnoses, management options, and outcomes. Curr Rev Musculoskelet Med.

[4] Goldberg AL, Kershah SM (2010). Advances in imaging of vertebral and spinal cord injury. J Spinal Cord Med.

[5] Blackmore CC (2003). Evidence-based imaging evaluation of the cervical spine in trauma. Neuroimaging Clin N Am.

[6] Parizel PM, van der Zijden T, Gaudino S (2010). Trauma of the spine and spinal cord: imaging strategies. Eur Spine J.

[7] Sundstrøm T, Asbjørnsen H, Habiba S, Sunde GA, Wester K (2014). Prehospital use of cervical collars in trauma patients: a critical review. J Neurotrauma.

[8] Rajasekaran S, Vaccaro AR, Kanna RM (2017). The value of CT and MRI in the classification and surgical decision-making among spine surgeons in thoracolumbar spinal injuries. Eur Spine J.

[9] Gamanagatti S, Rathinam D, Rangarajan K, Kumar A, Farooque K, Sharma V (2015). Imaging evaluation of traumatic thoracolumbar spine injuries: radiological review. World J Radiol.

[10] Small JE, Osler P, Paul AB, Kunst M (2021). CT cervical spine fracture detection using a convolutional neural network. AJNR Am J Neuroradiol.

[11] Langerhuizen DW, Bulstra AE, Janssen SJ, Ring D, Kerkhoffs GM, Jaarsma RL, Doornberg JN (2020). Is deep learning on par with human observers for detection of radiographically visible and occult fractures of the scaphoid?. Clin Orthop Relat Res.

[12] Kalmet PH, Sanduleanu S, Primakov S (2020). Deep learning in fracture detection: a narrative review. Acta Orthop.

[13] Zhou S, Zhou F, Sun Y (2022). The application of artificial intelligence in spine surgery. Front Surg.

[14] Voter AF, Larson ME, Garrett JW, Yu JJ (2021). Diagnostic accuracy and failure mode analysis of a deep learning algorithm for the detection of cervical spine fractures. AJNR Am J Neuroradiol.

[15] Boonrod A, Boonrod A, Meethawolgul A, Twinprai P (2022). Diagnostic accuracy of deep learning for evaluation of C-spine injury from lateral neck radiographs. Heliyon.

[16] Wei CJ, Tsai WC, Tiu CM, Wu HT, Chiou HJ, Chang CY (2006). Systematic analysis of missed extremity fractures in emergency radiology. Acta Radiol.

[17] Gale SC, Gracias VH, Reilly PM, Schwab CW (2005). The inefficiency of plain radiography to evaluate the cervical spine after blunt trauma. J Trauma.

